# Patient satisfaction with post-operative pain management and associated factors among surgical patients at Tikur Anbessa Specialized Hospital: Cross-sectional study

**DOI:** 10.1016/j.amsu.2022.104087

**Published:** 2022-07-02

**Authors:** Bekele Buli, Amanu Gashaw, Geresu Gebeyehu, Meron Abrar, Bayisa Gerbessa

**Affiliations:** a Department of Anesthesia, College of Medicine and Health Sciences, Hawassa University, Ethiopia; b Department of Anesthesia, College of Medicine and Health Sciences, Addis Ababa University, Addis Ababa, Ethiopia; c Department of Anesthesia, College of Medicine and Health Sciences, Dire Dewa University, Ethiopia

**Keywords:** Postoperative pain, Patients' satisfaction, Surgical patients

## Abstract

**Background:**

Patient satisfaction with postoperative pain management is a vital tool for measuring the quality of care in health centers, which associated with the care process and care outcome. There is still few evidence on factor for patient satisfaction with postoperative pain management.

**Objective:**

These study aimed to assess magnitude of Patient satisfaction with post-operative pain management and associated factors among surgical patients at Tikur Anbessa Specialized Hospital, from Feb 1- Apr 30, 2021.

**Method:**

Institutional based cross-sectional study was conducted among 335 adult patients using a systematic random sampling technique. Data were collected through structured questionnaires based on the modified APS-POQ to obtain responses from the patients. Both bivariable and multivariable logistic regression analysis was done to evaluate the association. P-value less than 0.05 was considered as statistically significant.

**Result:**

The find of this study revealed that 74.5% of patients were satisfied with overall pain management services. Patients with ASA I (AOR = 2.3; 95%CI: (1.06–5.08), received multimodal analgesics (AOR 4.30; 95% CI: (2.02–9.18), no perceived pain (AOR = 6.7; 95% CI: (1.54–29.7), had pain discussion (AOR = 8.9; 95% CI: (3.67–21.90) and waiting for analgesia service less than 30 min (AOR = 6.3; 95% CI: (1.34–29.58) were more satisfied.

**Conclusion:**

The study shows that patient satisfaction with postoperative pain management was low in our setup compared to many studies. Thus, there is a need to improve the quality of pain management services in the study area.

## Highlights

• Patient satisfaction with postoperative pain management was suboptimal.
• ASA status, pain, analgesic techniques & management were significantly associated.
• Patient satisfaction also associated with empathy, patient education, & communication.

## 1 Introduction

Postoperative pain is ‘acute pain due to surgical trauma with an inflammatory response and initiation of an afferent neuronal barrage that results in several unpleasant sensory, emotional and mental experiences’ [1].

Assessment of patient satisfaction becomes an important tool for health care services to measure outcome management. Patient satisfaction with postoperative pain management is the result of satisfaction with the care process and care outcomes, which include waiting time, provision of information, access and adequacy of care [2,3]. Patient satisfaction in health care settings generally encompasses both psychosocial and technical aspects of care, that strongly associated with effective pain management [4].

Suboptimal patient satisfaction with postoperative pain management is still remaining a common problem in health care [5]. Some studies reveal that satisfaction with postoperative pain treatment is less associated with patients’ actual pain experience, but rather with appropriateness of care and involvement in pain management [6]. The review of abroad literatures reported that conflicting arguments regarding patient satisfaction with pain management in contradict ways [[7], [8], [9]]. In spite of such studies, there is still limited clinical information available on the association of patient characteristics, patient perception of pain experience, and patient satisfaction with postoperative pain management [10].

Prior studies within our county related to postoperative pain management were mainly focused on the prevalence of postoperative pain intensity. During our search, there was few evidence that shows the magnitude and associated factors for patient satisfaction with postoperative pain management in the surgical patients of the study area. The available evidences in different set-up and populations will possibly affect the magnitude and associated factors of patient satisfaction.

This study might be helping the health service management to understand the magnitude of the problem and to highlight the awareness of the problem areas by concerned bodies. It might play an important role in this case as a baseline for next researches to be done in this area, to resolve the problems of patient dissatisfaction. The study also will give an insight on what the current status of satisfaction with postoperative pain management in our setup looks like.

## 2 Materials and methods

### 2.1 Study design, setting and population

Institutional based cross-sectional study design was conducted from February to April 2021 at TASH in Addis Ababa, Ethiopia. In the target hospital, it has been estimated that 2500- 3000 adult elective surgeries were operated each year. This work is reported in line with STROCSS criteria from www.strocssguideline.com [11]. To date, postoperative pain was controlled according to national guidelines, particularly using pethidine, tramadol, and diclofenac prescribed by the surgeons and provided by nurse wards at study area [12]. The study included all adult patients who had undergone elective surgery during the study period at TASH. Patients who were critically ill and unable to communicate and postoperative admission in the intensive care unit were excluded from this study.

### 2.2 Sample size and sampling technique

Sample size was determined by a single population proportion formula with a previous study done in Gondar Hospital (72.2%) and the assumptions were made: level of confidence 95%; Zα/2 = 1.96, 5% margin of error (d = 0.05) [13].

$$SS=\frac{\left(\mathrm{Z\alpha}\right)²\text{ p}\left(1-\text{p}\right)}{\text{d}2}$$

where, Zα is p = 0.723, 1-p = q = 0.277, d = 0.05

$$n\;=\frac{\left(1.96\right)²\left(0.723\right)\left(0.277\right)}{\left(0.05\right)²}\;=307$$

Thus, the calculated sample size and adding for 10% a possible nonresponse rate resulted in a total sample size of 338 patients. Situational analysis from the operation log book has been shown that 750 elective adult surgeries were done in the last 3 months, and 338 participants are recruited with the probability of K^th^ = 750/338 ∼ every 2 patients. The total sample size was selected by using a systematic random sampling technique at every K interval using the registration list from the recovery room as a sampling frame among postsurgical patients.

### 2.3 Study variables [11]

**Dependent Variable:** Level of patient satisfaction with postoperative pain management: Satisfied or Dissatisfied.

**Independent Variables:** Socio demographic factors, clinical related factors, and pain management related factors.

### 2.4 Data collection procedure

The Questionnaires were adapted from Revised American Pain Society Patient Outcome Questionnaire (APS-POQ) and modified to align with the study objectives [14]. The study questionnaire consisted of two sections: The first section had questions regarding the participant's demographic details, including age, gender, level of education & clinical characteristics. The second section used the APS Patient Outcome Questionnaire (APS-POQ), which asks about the patient's pain experience, including: 1) pain intensity within the past 24 h using a scale of 0 (no pain) to 10 (severe pain); 2) pain interference with daily activities and current pain on the scale; 3) waiting time for pain medication, 4) satisfaction with 5 aspects of pain management using a 5-item Likert scale ranging from 0 (very dissatisfied) to 5 (very satisfied) [15].

Three anesthesia students of AAU University School of Medicine were properly trained to collect the required data. Data were collected through patients' chart review and interviewing. The modified APS-POQ-R survey contained 10 questions asked about the patient's postoperative pain experience, which were translated to Amharic language and translated back to English by language experts. Pretest was done at Minilik hospital on 34 patients (10% of the estimated sample size) and some amendment was done before the actual data collection. During the study, it was determined that certain survey questions were confusing to patients; thus, a few changes were made to the survey for use in this study. Minor changes were made to some words which seemed to reflect the same concepts to study participants such as worst pain and pain interfere with their daily activities, thus, the question was changed to “… the worst amount of pain interfere with you stay asleep …“. The postoperative pain management modalities used (i.e., systemic analgesics, and regional techniques) were noted, but the doses of analgesics were not recorded. Pain intensity was measured based on verbally responded numerical rating scales (VNRS) with answer options ranging from 0 to 10, where 0 reflects no pain, 1–3 mild, 4–6 moderate, and 7–10 severe pain [15].

### 2.5 Data quality control

The study was approved by the AA University Institutional Review Board and the applicable executives of the involved hospitals. The study was performed in accordance with the ethical standards as laid down in the 1964 Declaration of Helsinki and its later amendments. The quality of data was ensured before, during, and after data collection. Orientation about the objectives and relevance of the study, each item included in the study tools, and the whole process of data collection was provided for data collectors. Informed consent was obtained from data collectors. During data collection, regular supervision and follow-up was undertaken. Supervisors were checking each questionnaire daily with further cross-check by the principal investigator for completeness and consistency of data. Data clean-up and crosschecking of missing data was done by multiple imputation method before analysis with SPSS.

### 2.6 Data analysis and interpretation

Data was entered into SPSS version 26.0 for analysis. The frequency, percentage, and cross-tabulation of different variables were determined. Models of fitness were checked by Hosmer Lemeshow goodness-of-fit test and the magnitude and associated factors were analyzed using binary logistic regression and multivariable logistic regression. Variables with P-value < 0.2 binary logistic regression were included in a multivariable logistic regression. Finally, the p value of 0.05 and less was considered as statistically significant. The AOR was used to determine the strength of the association between a dependent and independent variable. Satisfaction through ‘five-point Likert scale was dichotomized in to satisfied and dissatisfied groups based on demarcation threshold formula:’ = $\frac{\left(\text{total highest score }-\text{ total lowest score}\right)}{2}+total\;lowast\;scores$ [16].

## 3 Results

### 3.1 Socio demographic and clinical characteristics of participants

A total of 335 patients participated in the study with a response rate of 99.1%. Three patients were excluded from the analysis for incomplete data. Most of the respondents, 137 (40.9%) were in the mid-age group with the mean age ± SD being 41.5 ± 8.51 years. 177 (52.3%) were female and 107(31.8%) had not received any formal education. Majority of the study participants 68.2% were ASA I status.

### 3.2 Clinical characteristics of study participants

Of the total, the majority of patients (60.3%) were undergoing surgery under general anesthesia while 39.7% were operated under regional anesthesia (see Table 1). The distributions of surgical procedures were (50.7%) abdominal surgery followed by limb surgery (36.7%) of participants and the remaining 12.5% were head and neck surgery. In the perioperative period, 192 (57.4%%) of patients received multimodal analgesia with regional block. Post-operatively, 256(76.4%) of the study participants were experienced at least mild to severe pain (Table 2, Table 3).

**Table 1:** Socio, demographic, and clinical characteristics of patients who underwent surgery at TASH, Addis Ababa, Ethiopia, 2021(*N* = 335).

Variables	Categories	Frequency (*n*)	Percentages (%)
Gender	Male	158	47.2
	Female	177	52.8
Age*	18–35	115	34.3
	36–55	137	40.9
	55+	83	24.8
Education	Illiterate	107	31.8
	Literate	218	68.2
ASA status	ASA1	228	68.2
	ASA2	76	22.7
	ASA3	28	8.4

**Table 2:** Clinical related characteristic of patients who underwent elective surgery at TASH from Feb to Apr 30, 2021. (n = 335).

Variables	Categories	Freq.	Percent (%)
Site of surgery	Limbs	123	36.7
	Head and neck	42	12.5
	Upper abdomen	67	20.1
	Lower Abdomen	103	30.7
Types of anesthesia	GA	202	60.3
	SA	133	39.7
Analgesia modality	Systemic analgesia	143	42.6
	Multimodal	192	57.4
Postoperative pain score (VNRS)	VNRS(0)	78	23.6
	VNRS(1–3)	120	35.8
	VNRS(4–6)	85	25.4
	VNRS(7–10)	52	15.5

**Table 3:** Bi-variable logistic regression analysis of patient satisfaction with post-operation pain management at TASH, Addis Ababa, May 2021 (n = 335).

Variables	Categories	Satisfied N (%)	Dissatisfied N (%)	COR(95% CI)	P value
Age	18–35	63(58.5)	42(41.5)	1.12(.58–2.14)	.07
	36–55	83(67.6)	36(32.4)	1.1(.54–2.14)	.97
	55+	50(74.1)	28(25.9)	1	1
Gender	M	118(66.1)	38(33.9)	0.62(.37–1.02)	.26
	F	116(72.4)	60(27.6)	1	
Education	Illiterate	83(67.8)	33(32.2)	1.6(.97–2.9)	.06
	literate	151(75.7)	55(24.3)	1	
ASA status	ASA I	182(78.3)	64(22.7)	2.2(1.32–3.64)	**.002**
	ASA II&III	67(72.3)	40(27.37)	1	
Analgesia technique	Systemic	104(67.0)	61(37.0)	1	
	Multimodal	145(91.2)	25(8.8)	5.3(3.03–9.29)	.000*
Anesthesia types	GA	143(80.2)	57(19.9)	1.12(0.68–1.8)	**0.6**
	RA	91(65.4)	41(34.5)	1	
Surgery types	Limbs	83(68.2)	38(31.8)	0.5(0.3–1.2)	.15
	Lower abdomen	86(75.7)	26(24.3)	2.9(1.3–6.7)	**.01**
	Upper abdomen	56(64.7)	20(36.3)	1.18(0.6–2.1)	.16
	Head & neck	25(50.5)	24(49.5)	1	1
Pain score	VNRS(0)	72(81.7)	6 (28.7)	26.4(8.3–83.25)	
	VNRS(1–3)	95(73.6)	25(26.3)	5.3(2.6–10.83)	.000*
	VNRS(4–6)	47(52.4)	38(47.6)	1.76(0.85–3.64)	.000*
	VNRS(7–10)	24(48.5)	28(51.5)	1	.124
Waiting time (in minutes)	Less than 30	192(88.6)	24 (11.4)	6.9(2.64–18.03)	.000*
	More than 30	57(48.9)	61 (51.1)	1	
Discuss pain	Yes	154(96.2)	16 (3.8)	11.78(5.8–23.8)	.000*
	No	95(54.6)	75 (45.4)	1	

*P value < 0.001, 1 = reference group, COR = crude odds ratio.

### 3.3 Overall satisfaction with postoperative pain management

**Fig. 1:**
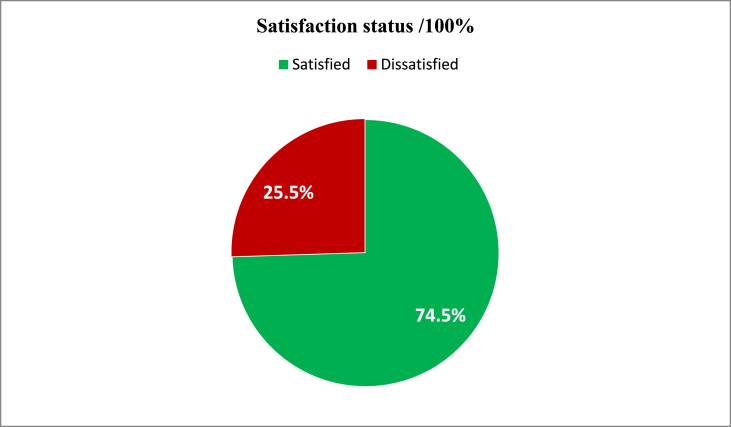
Overall patient satisfaction with postoperative pain management of elective adult patient at TASH, May 2021.

In this study, the variables used to measure the overall level of satisfaction and pain management, and determine the overall level of satisfaction when the responses were dichotomized into satisfied and dissatisfied. Respondents who scored mean and above were categorized as satisfied and those who scored below were categorized as dissatisfied. Hence, 278(74.5%) study participants were satisfied with their pain management and 107(24.5%) were dissatisfied with details, see Fig. 1.

### 3.4 Factors associated with Patient's satisfaction with pain management

Binary logistic regression analysis was done to evaluate the presence of association between independent variables and dependent variables (overall satisfaction). Among those variables; age, educational status, disease status, surgery type, pain intensity, analgesia modality, waiting time to get analgesia, and whether they told reporting pain (pain communication) were found to be significantly associated with overall satisfaction at p-value <0.2.

### 3.5 Multivariable logistic regression with overall satisfaction

In the multivariable logistic regression analysis, ASA status, pain severity, analgesic modality, waiting time, getting adequate information on pain, were significantly associated variables for patient satisfaction with pain management services. The main promising finding was that participants who were informed on reporting pain management were 8.9 times satisfied (AOR 8.97; 95%CI: 3.6–21.90) than those who were not informed as shown in Table 4.

**Table 4:** Multivariate analysis results of patient satisfaction with postoperative pain management assessed at TASH (N = 335).

Variables	Descriptive	COR(95%CI)	AOR (95% CI)	P Value
ASA status	ASA I ASA II& III	2.2(1.32–3.64) 1	2.33(1.07–5.08) 1	**0.03**
Pain score (VNRS)	VNRS(0)	26.4(8.37–83.25)	6.7(1.54–29.7)	**0.001**
	VNRS(1–3)	5.3(2.6–10.83)	4.5(1.65–12.10)	**0.03**
	VNRS(4–6)	1.76(.85–3.64)	1.44(.516–4.021)	0.48
	VNRS(7–10)	1	1	
Analgesia modality	Systemic	1	1	
	Multimodal	5.3(3.03–9.29)	4.30(2.02–9.18)	**P<0.001**
Waiting time (in minutes)	Less than 30	6.9(2.64–18.03)	6.30 (1.34–29.5)	**0.001**
	More than 30	1	1	
Pain discussion	Yes	11.78(5.81–23.8)	8.97(3.68–21.90)	**P<0.001**
	No	1	1	

1 = reference group, COR = crude odds ratio, AOR = adjusted odds ratio, CI = confidence interval.

## 4 Discussion

The overall result of this study showed that 74.5% participant was satisfied with postoperative pain management. This result showed that there was a slight improvement in patient satisfaction compared to a prospective study conducted in Jimma by W. Esthete and the study at the Gondar specialized hospital that showed that only 50.0% and 72.2% of the study participants respectively were satisfied with their management, this might be due to time and working setup difference between the study participants [13,17,18].

This finding was also low compared with other studies in Malaysia, Pakistan, Ghana, and Tanzania [[19], [20], [21], [22]]. The reason of this finding might be due to the difference between pain management techniques/strategies like; good general caring attitude of pain management service teams, high rate of pain education, and good communication or superior use of analgesia or demographic characteristics as explained in those studies.

In our study, socio-demographic factors, age, sex, and education level were not significantly associated with the level of patient satisfaction. However, a study conducted by Tawil et al., in 2018 showed older ages were more satisfied than middle age group, and Subramanian et al. female participants were more satisfied than male participants and less educated people were high satisfaction level [23,24]. This variation may be a subjective and complex concept of patient satisfaction.

Regarding factors associated with patient satisfaction to postoperative pain management, five variables were statistically identified. The first patients in ASA group were 2.3 times more likely satisfied with pain management (AOR; 2.33(1.07–5.08), P = 0.03. In agreement with this study, the study done by Josef et al. at Gondar reported that ASA I status was associated with good satisfaction, 3.5 times more likely to be satisfied compared with other groups of patients (AOR = 3.55 (1.20–10.55) [13]. Another study conducted in Pakistan also supports our study, where ASA I patients were 3.7 times more likely to be satisfied compared with other groups [25].

Secondly, the study showed lower mean pain scores (AOR: 6.7:95% CI; (1.54–29.7) resulted in higher satisfaction levels. This finding is comparable to previous studies that found decreased patient satisfaction with increased pain scores [[26], [27], [28]]. The studies reveal the negatively associated factors of patient satisfaction with the pain experienced; thus, the more pain intensity, the lower the satisfaction level. In the other way, this finding is incongruent with studies showing that patients could be pleased with their pain management despite experiencing severe pain [15,29]. The reason for satisfaction might be unrealistic expectations for appropriateness of care rather than actual pain experience.

Thirdly, analgesic techniques were another strong associated factor of satisfaction. Our finding shows that multimodal analgesia of nerve block recipients were 4 times (AOR: 4.30; 95% CI; (2.02–9.18) associated with a high level of satisfaction. It is consistent with other studies that reveal patients taking postoperative nerve block were 9 times more likely to be satisfied compared with patients without nerve block [25,30]. It might be due to the administration of multimodal analgesia drugs which would be expected to decrease pain scores considerably, thereby increasing patient satisfaction and the fact that the regional block has superior analgesia for pain management. This finding is also congruent with the results derived from different studies which reported a higher rate of patient satisfaction with multimodal analgesia recipients [21,24].

Fourthly, the positively associated patient satisfaction with postoperative pain management were related to patient engagement in the care process to ensure good communication. This study demonstrated that the recipients of specific pain communication were 8.9 times satisfied than those who did not get involved in pain management decisions (AOR: 8.9: 95% CI; (3.68–21.90). This might be due to participants that had enough information about pain management and able to discuss their fears were more likely to be satisfied compared to those patients who did not get it. This result was also in line with the study done by Botti et al. and Schwenkglen e.t. al, which suggested that the patient satisfied with pain management affected by good communication [6,31].

Lastly, in this study, waiting a short time to respond to their pain (AOR = 6.3; 95%CI (1.34–29.5) were positively associated with satisfaction. This finding is comparable to previously reported results that waiting a long time decreases the probability of being satisfied [20,21]. This is also congruence with the study in Lebanon by Tawil et al. that shows the fact that patients wait for more than 30 min before getting the pain medication requested and did not get any additional analgesics for pain relief were negatively associated with patient satisfaction [23]. This finding is comparable to previously reported in India and Malaysia results that waiting a long time decreases the probability of being satisfied with overall postoperative pain management [20,21].

### 4.1 Limitation of study

✓ The dichotomized Likert data might lead to loss of information about satisfaction status due to unequal distance space
✓ The study did not include critically ill patients; this issue might affect dependent variables, so our findings were interpreted with these limitations.

## 5 Conclusion and recommendations

The study revealed that patient satisfaction with postoperative pain management was suboptimal. Associated factors with patient's satisfaction with postoperative pain management such as: ASA status, postoperative pain intensity, analgesic techniques used, and management process were identified significantly. Patient satisfaction with postoperative pain management is not only based on the presence or absence of pain but also on provider empathy, patient education, and provider communication on pain management. To improve patient satisfaction, attention should be paid to achieving acceptable pain levels, providing patients with helpful information about their pain treatment, and, allowing patients to participate in decisions about their pain management and highlight the need for timely provision of pain management.

## Ethical approval

Ethical clearance to conduct the research was obtained from the Ethical Review Committee of the School of Medicine, College of Medicine and Health Sciences, Addis Ababa University.

## Consent

Written informed consent was obtained from each study participant after a clear explanation of what they would have to do and take part in the study. Anyone not willing to participate in the study was informed that they have the full right not to participate or stop at any time and those who were not voluntary were excluded. Confidentiality was guaranteed by keeping the secrecy of personal identification, keeping the completed questionnaires and checklist results in a well-secured area.

## Registration of research studies

1. Name of the registry:
2. Unique Identifying number or registration ID:
3. Hyperlink to your specific registration (must be publicly accessible and will be checked):

## Guarantor

All authors.

## Data availability

All data generated or analyzed during this study are included within this article.

## Declaration of competing interest

All authors declare that they have no conflicts of interest.

## Abbreviations

**APOP**: Acute Post-operative Pain
AOR: Adjusted odds ratio
**ASA**: American Society of Anesthesiologists
**IASP**: International Association for the Study of Pain
**NRS**: Numeric Rating Scale
**PCA**: Patient-Controlled Analgesia
**POD**: Postoperative Days
**TASH**: Tikur Anbessa Specialized Hospital
**VRS**: Verbal Rating Scale
